# Protective effect of salidroside against bone loss *via* hypoxia-inducible factor-1α pathway-induced angiogenesis

**DOI:** 10.1038/srep32131

**Published:** 2016-08-25

**Authors:** Ling Li, Ye Qu, Xin Jin, Xiao Qin Guo, Yue Wang, Lin Qi, Jing Yang, Peng Zhang, Ling Zhi Li

**Affiliations:** 1 Tianjin Key Laboratory for Prevention and Control of Occupational and Environmental Hazard, Tianjin, People’s Republic of China; 2 Department of Pharmacology, Logistics College of Chinese People’s Armed Police Forces, Tianjin, People’s Republic of China; 3 Department of Pathogenic Biology and Immunology, Logistics College of Chinese People’s Armed Police Forces, Tianjin, People’s Republic of China; 4 Department of Orthopaedics, Affiliated Hospital of Logistics College of Chinese People’s Armed Police Forces, Tianjin, People’s Republic of China; 5 Department of Pharmaceutical Chemistry, Logistics College of Chinese People’s Armed Police Forces, Tianjin, People’s Republic of China

## Abstract

Hypoxia-inducible factor (HIF)-1α plays a critical role in coupling angiogenesis with osteogenesis during bone development and regeneration. Salidroside (SAL) has shown anti-hypoxic effects *in vitro* and *in vivo*. However, the possible roles of SAL in the prevention of hypoxia-induced osteoporosis have remained unknown. Two osteoblast cell lines, MG-63 and ROB, were employed to evaluate the effects of SAL on cell viability, apoptosis, differentiation and mineralization *in vitro*. Rats subjected to ovariectomy-induced bone loss were treated with SAL *in vivo*. Our results showed that pre-treatment with SAL markedly attenuated the hypoxia-induced reductions in cell viability, apoptosis, differentiation and mineralization. SAL down-regulated HIF-1α expression and inhibited its translocation; however, SAL increased its transcriptional activity and, consequently, up-regulated vascular endothelial growth factor (VEGF). *In vivo* studies further demonstrated that SAL caused decreases in the mineral, alkaline phosphatase (ALP), and BGP concentrations in the blood of ovariectomized (OVX) rats. Moreover, SAL improved the trabecular bone microarchitecture and increased bone mineral density in the distal femur. Additionally, SAL administration partially ameliorated this hypoxia *via* the HIF-1α-VEGF signalling pathway. Our results indicate that SAL prevents bone loss by enhancing angiogenesis and osteogenesis and that these effects are associated with the activation of HIF-1α signalling.

Osteoporosis, defined as low bone mass that leads to increased fracture risk, is a major health problem. Osteoporosis is a degenerative bone disease characterized by low bone mass and the structural deterioration of bone tissue, which result in bone fragility^1^. Hypoxia was reported to induce osteoblast apoptosis and to decrease osteoblast activity, leading to reduced osteoblastic bone formation^2^. Moreover, bone loss often occurs at sites of hypoxia, and decreased blood supply to bone tissue ultimately leads to a reduction in bone mass^3^. Previous studies have shown that blood vessels are important in bone formation, as they are key contributors to the process of osteogenesis during bone development and repair^4^,^5^.

Angiogenesis depends on hypoxic stimuli and vascular endothelial growth factor (VEGF) production. The hypoxia-inducible factor (HIF)-1α pathway is the central regulator of the adaptive response to low oxygen levels. This pathway regulates angiogenic genes (e.g., VEGF, angiopoietins)^6^. Wang *et al*. showed that activating the HIF-1α pathway in mature osteoblasts in developing bone increased bone modelling; this effect was predominantly due to enhanced VEGF-mediated bone vessel formation^7^. Similar results were reported by Rankin EB *et al*., who generated mice in which HIF-1α was over-stabilized in osteoprogenitor cells. The mice exhibited excessive accumulation of trabecular bone in long bones and increased vascularization^8^. These data are consistent with the findings of Zhao Q *et al*., who demonstrated that bone density, bone vessels, and bone formation activity were lower in HIF-1α conditional knockout (KO) ovariectomized (OVX) mice than in wild-type OVX mice^9^. Moreover, the expression of HIF-1α and VEGF was decreased in OVX mice but not in *Vhl* KO OVX mice. In addition, *Vhl* KO OVX mice, which show increased angiogenesis and osteogenesis due to activation of the HIF-1α pathway, were protected from OVX-induced bone loss^10^. Consequently, the HIF-1α pathway is a critical mediator of neoangiogenesis, which is required for skeletal regeneration. This finding suggests the application of HIF activators as therapeutics to improve bone healing.

Salidroside (SAL) is a major biologically active compound extracted from the root of *Rhodiolarosea* L. This compound has long been used in traditional Chinese medicine. Previous reports have indicated that SAL possesses diverse pharmacological effects, including anti-hypoxic, pro-angiogenic and anti-osteoporotic activities^11^,^12^,^13^. Furthermore, recent studies have indicated that SAL protects cardiomyocytes against hypoxia-induced death *vi*a HIF-1α and VEGF-mediated pathway^14^. However, whether SAL could provide protection against bone loss by promoting osteogenesis *via* the enhancement of angiogenesis remained unknown. Therefore, in this study, we employed an *in vitro* CoCl_2_-induced hypoxia model using human osteoblastic MG-63 cells and osteoblast primary cultures, which are commonly used to study osteogenic development, and an *in vivo* ovariectomy-induced osteoporosis rat model to investigate the protective effects of SAL against bone loss. Furthermore, the mechanism by which SAL alters HIF-1α pathway activity was investigated.

## Materials and Methods

All experimental procedures were approved by the Institutional Animal Care and Use Committee and the Tianjin Hospital Ethics Committee of China. The methods were carried out in accordance with the approved guidelines.

### Materials

SAL extracted from *Rhodiolarosea* L (purity 99%) was obtained from the National Institute for the Control of Pharmaceutical and Biological Products (Beijing, China). The alkaline phosphatase (ALP) activity assay kit was purchased from Jian cheng Biotechnology Institute (Nanjing, China). Osteocalcin and type I collagen enzyme-linked immunosorbent assay (ELISA) kits were purchased from Senxiong Technologies, Inc. (Shanghai, China). The human VEGF ELISA kit was obtained from Sizhengbai Technologies, Inc. (Beijing, China). The annexinV/propidiumiodide (PI) apoptosis assay kit was purchased from Bio Legend Co. (USA). Anti-β-actin antibody was purchased from Santa Cruz Biotechnology, Inc. (USA). Anti-HIF-1α and anti-pVHL antibodies were purchased from Abgent Biotechnology, Inc. (USA). Anti-Osterix and anti-Runx2 antibodies were purchased from Abcam Biotechnology, Inc. (USA). FITC-labelled fluorescent secondary antibodies were purchased from KPL Co. (USA). DAPI was obtained from Roche Applied Science (Germany). The Diagnostic Acid Phosphatase kit (Sigma-Aldrich) for tartrate-resistant acid phosphatase (TRAP) staining.

### Cell lines

Human osteoblastic MG-63 cells were obtained from American Type Culture Collection (Rockville, MD, USA). ROB cells were isolated from 0- to 5-day-old Sprague Daw ley rats *via* enzymatic digestion as previously described^15^. Briefly, the parietal and occipital bones were dissected and minced. Then, 700 U/ml type I collagenase (C0130, Sigma-Aldrich, St. Louis, MO, USA) was used to release the ROB cells from the bone, and the first supernatant was discarded.

### MTT assay

In this experiment, 4 × 10^3^ cells per well were seeded in 96-well plates overnight. Then, the cells were cultured in medium containing 1% charcoal-stripped FBS (sFBS) (HyClone Laboratories, Inc.) for 24 h. Cells were treated with standard 1% FBS culture medium containing SAL (0, 1, 10, 100, or 1000 nM) in DMSO for 24 h, followed by treatment with CoCl_2_ for 24 h. Cell viability was measured by MTT assay.

### Flow cytometric assay

The percentage of apoptotic cells was measured using an annexin V/PI apoptosis assay kit, and the samples used for flow cytometric analysis were prepared according to the manufacturer’s instructions. The cells were treated with SAL (10 or 100 nM), followed by treatment with CoCl_2_ or vehicle (DMSO). Next, the cells were collected and washed twice each with cold PBS and cold Bio Legend cell staining buffer. The cells were resuspended in AnnexinV Binding Buffer at a concentration of 1 × 10^6^ cells/ml. Next, 100 μl of cell suspension was transferred to a 5 ml test tube, and 5 μl of annexinV-FITC and 10 μl of PI solution were added. The cells were gently vortexed and then incubated for 15 min at room temperature (25 °C) in the dark. Afterwards, 400 μl of AnnexinV Binding Buffer was added to each tube, and the cells were analysed using a flow cytometer (Beckman-Coulter Inc., USA).

### Semi-quantitative RT-PCR

Total RNA was isolated from cells using TRIzol (Invitrogen) according to the manufacturer’s instructions. Primer sequences were designed using Vector NTI 8 software and were synthesized by TaKaRa Biotechnology Co., Ltd. (Dalian, China). A One Step RNA PCR Kit (AMV) (TaKaRa BiotechnologyCo., Ltd.) was used to perform RT-PCR. The PCR products were subjected to electrophoresis on a 1.5% agarose gel, which was analysed using Quantity One version 4.5.6 software (Bio-Rad, Hercules, CA, USA). The results were normalized to the mRNA levels of β-actin and were presented as the ratio of target mRNA to β-actin mRNA.

### Western blot analysis

Cells were collected and resuspended in lysis buffer. After 30 min on ice, the lysates were centrifuged at 12000 × *g* at −20 °C for 30 min, and the protein concentration was determined using the bicinchoninic acid method (BCA kit, Applygen Technologies Inc., Beijing, China). Protein samples containing 30–80 μg of total protein were separated on 10–12% gels *via* SDS-PAGE and were analysed *via* Western blot using mouse monoclonal anti-HIF-1α, rabbit polyclonal anti-pVHL, rabbit monoclonal anti-Runx2 and rabbit polyclonal anti-Osterix antibodies. The membranes were stripped *via* incubation in stripping buffer at 50 °C for 30 min. Then, the membranes were probed with a mouse monoclonal anti-β-actin antibody. Immunodetection was performed using the corresponding secondary HRP-conjugated antibody, and HRP activity was detected using a chemiluminescent substrate kit (SuperSignal^®^ Westpico Trial Kit, Pierce Biochemicals).

### Measurement of ALP activity

MG-63 or ROB cells were seeded in 12-well plates at a density of 2 × 10^5^ cells per well. The following day, the cells were placed in 1% FBS-containing culture medium for 24 h and then treated with the same culture medium containing SAL (0, 1, 10, 100, or 1000 nM) for 2, 4, or 6 days, followed by treatment with CoCl_2_. For harvesting, the cells were washed three times with PBS and incubated in 0.1% Triton X-100 for 3 min. After sonication, the lysate was centrifuged at 1500 rpm for 15 min. To assess ALP activity, the absorbance of the clear supernatant at 520 nm was measured. One unit of ALP activity was defined as the activity that produced 1 mg of phenol, and ALP activity was expressed as U/100 ml of sample. Relative ALP activity was estimated by dividing the ALP activity level (in U/100 ml) by the absorbance of MTS at 490 nm.

### ELISA

The cells were cultured for 24 h in 1 ml of medium containing 1% FBS and then treated with different concentrations (0, 1, 10, 100 or 1000 nM) of SAL in DMSO for 2, 4, or 6 days, followed by treatment with CoCl_2_. The supernatants were collected and clarified *via* centrifugation. The levels of type I collagen, osteocalcin and VEGF (only at 2 days) were determined *via* ELISA (Shanghai Senxiong Technologies, Inc., China) according to the manufacturer’s instructions. The relative type I collagen and osteocalcin levels were estimated by dividing the type I collagen (ng/ml) and osteocalcin concentration (ng/ml), respectively, by the absorbance of MTS at 490 nm.

### Bone nodule formation

ROB cells were seeded in 6-well plates at a density of 4 × 10^5^ cells/ml and cultured as described above for 24 h. The cells were then incubated in medium containing 2% FBS for an additional 24 h. Next, the cells were treated with SAL (10 or 100 nM), followed by treatment with CoCl_2_ or vehicle (DMSO). Once the cells reached 50% confluence, ascorbic acid (100 μg/ml) and beta-glycerol phosphate (10 mmol/l) were added to the cultures. The treated medium was replenished every 2 days. After 18 days in culture, the cells were fixed in 95% ethanol for 30 min and stained with 0.1% Alizarin Red S Tris-HCL (pH 8.3, Sigma) for 30 min at 37 °C. To estimate matrix calcification, the stain was solubilized with 10% cetylpyridinium chloride (pH 7.0) by shaking for 15 min. The absorbance of the released Alizarin Red S was measured at 570 nm.

### Immunofluorescence and confocal microscopy

Briefly, MG-63 cells were plated on lysine-treated glass coverslips placed in 6-well plates. After the cells were cultured for 24 h under either normoxic or hypoxic conditions, they were washed three times with PBS, fixed with 4% paraformaldehyde for 10 min at room temperature, permeabilized with 0.5% Triton X-100 for 10 min (washed three times with PBS), and blocked with blocking solution (3%BSA) for 60 min. Subsequently, the cells were incubated overnight at 4 °C in anti-HIF-1α antibody at a dilution of 1:1000. After the cells were washed, the coverslips were incubated in FITC-labelled goat anti-rabbit IgG (H + L) secondary antibody for 2 h in the dark and were mounted with VECTASHIELD mounting medium containing DAPI for 4 min in the dark. The location of fluorescence was detected by immunofluorescence and confocal microscopy (TCS SP5, Leica). Images were obtained using identical confocal microscopic settings for each set of experiments; no auto fluorescence was detected using these settings. Controls in which primary or secondary antibodies were omitted showed no staining.

### Transient transfection and luciferase reporter assay

Transfection was performed using Lipofectamine^TM^ 2000 (Invitrogen) according to the manufacturer’s instructions. MG-63 cells were cultured in 12-well plates until 90–95% confluence, followed by transfection. The cells were transfected with 2 μg of DNA containing a luciferase reporter gene linked to the proximal region of the HIF-1α promoter pGL2-HIF-1α (228 bp) or to two copies of a consensus HRE cloned upstream of the minimal herpes simplex virus thymidine kinase promoter (HRE2-TK-Luc) and with 1μg of β-galactosidase DNA, which was included to evaluate transfection efficiency. After 24 h, the cells were treated in 1% sFBS containing CoCl_2_ (0.5 mM) with or without SAL (10 or 100 nM) in the presence of PMA (200 nM final concentration, Sigma) for 24 h. Then, the cells were lysed, and luciferase activity was measured using the Luciferase Assay System (Promega, Madison, WI) and recorded using a Thermo Fluoroskan Ascent FL microplate fluorometer and luminometer (Thermo Electron Corporation). Additionally, β-galactosidase activity was detected using ONPG substrate (Sigma) and measured at 420 nm using an ELISA micro plate reader. The luciferase activity levels were normalized to the β-galactosidase activity levels and were presented as the mean relative luciferase activity of three independent experiments.

### Animals and SAL treatments

Twelve-week-old 230–270 g female Wistar rats were purchased from the Experimental Animal Centre of the Academy of Military Medical Sciences (Beijing, China) and were acclimated to the laboratory conditions for 1 week before the experiment. The initial body weights of the rats did not significantly differ between the five groups examined in this study. The animals were maintained in a well-ventilated controlled room at 20 °C on a 12 h light/dark cycle with free access to water and food. Rats underwent sham operation (n = 9) or were surgically OVX (n = 36) under anaesthesia with diethyl ether. Ovariectomy was performed by removing the bilateral ovaries through a dorsal approach, and sham surgery was performed by only identifying the bilateral ovaries. The rats were randomly divided into five groups: (1) untreated (Sham: sham-operated control), (2) untreated (ovariectomy-only control/model group), (3) SAL-L (4 mg/kg bodyweight), (4) SAL-H (20 mg/kg body weight), and (5) raloxifene (RLX group, 4 mg/kg body weight). SAL was dissolved in normal saline (NS), and NS alone was administered to untreated rats. The treatments were initiated 1week after the surgery and lasted for 12 weeks. Additionally, the rats were administered 30 mg/kg tetracycline *via* intraperitoneal injection 10 and 3 days before they were sacrificed to evaluate the bone mineral apposition rate (MAR). Moreover, the rats were administered 30 mg/kg Hypoxyprobe-1 *via* intraperitoneal injection 1 day before they were sacrificed to evaluate hypoxia in bone tissue. Blood samples were collected from the femoral artery, and serum was subsequently prepared *via* centrifugation. The right femur from each rat was removed and cleaned of adherent tissue. All experimental procedures using animals were approved by the Ethics in Animal Research Committee of the Academy of Military Medical Sciences.

### Determination of the levels of serum minerals, ALP, BGP and E_2_

After the 12-week treatment period, the rats were anesthetized *via* intraperitoneal injection of 20% (w/v) urethane solution at a dose of 1 mg/kg. Blood samples were collected from the femoral artery, and serum was subsequently obtained *via* centrifugation at a low temperature of 4 °C. The serum levels of minerals were determined using an automated biochemical analyser. ALP activity was determined using an Alkaline Phosphatase Opt Kit (Roche Molecular Biochemical, Indianapolis, IN, USA). The BGP and E_2_ levels in serum were measured *via* ELISA using rat-specific ELISA kits (Biomedical, Wien, Austria). All measurements were performed according to the manufacturer’s instructions.

### Bone mineral density (BMD) and femur weight

After removing soft tissues, the BMD of the right femurs was measured *via* dual-energy X-ray absorptiometry using a detector that was attached to small animal measurement software (Hologic, Bedford, MA, USA). The bones were cleaned of soft tissue, and the length and the wet weight of the bones were recorded. In addition, the femurs were dried to a constant weight at 120 °C. After adaptation to room temperature, the dry weight of each bone was recorded. Finally, the dry femurs were subjected to ashing at 800 °C for 2 h, and the ash weight was recorded.

### Biomechanical parameter analysis

The mechanical properties of the femurs were determined *via* the classical three-point bending test using an INSTRON 5865 (Instron Corporation, USA) with the following parameters: 20 mm gauge length, 200 N preload and 6 mm/min loading rate. Parameters related to structural mechanics (fracture displacement, fracture load, fracture energy, yield displacement, yield load, and yield energy) and material mechanics (modulus of elasticity, fracture stress and fracture strain) were recorded.

### Histomorphological analysis

Histomorphological analysis was performed to observe the changes in bone tissue. The right femurs were fixed in 10% buffered formalin for 2 days, decalcified in 14% ethylenediaminetetraaceticacid for 2 weeks, embedded in paraffin, and sliced into 5 μm thick coronal sections. The sections were stained with Giemsa, von Kossa stains and TRAP staining to evaluate the changes in bone. Static and dynamic histomorphometric measurements of the secondary trabecular bone located within 4.0 mm distal to the metaphyseal growth plate were performed. The specimens stained with von Kossa stain were used for histological observations, and the micromorphological parameters of trabecular bone in the femur, including percent trabecular area (Tb.Ar), trabecular thickness (Tb.Th), trabecular separation (Tb.Sp) and trabecular number (Tb.N), were analysed using Image-Pro Plus imaging software (Media Cybernetics, Silver Spring, MD, USA). In addition, we quantified micromorphological parameters of osteoid, including osteoid surfaces (OS), osteoid width (O.Wi) and osteoid volume (OV), using the same software. Alternatively, the specimens stained with Giemsa stain were used to observe osteoblast absorption surfaces (Ob.S). MAR measurements were recorded in regions of the specimens in which double labelling was detected. The width of the newly mineralized bone layer at the surface of the trabeculae is reported in terms of the MAR, which was expressed in units of microns per day, as determined by measuring the distance between the midline of two parallel fluorescent labels^16^. These measurements were performed using Image-Pro Plus software. The MAR was calculated for the cortical bone layer of the femur.

### Immunohistochemical analysis

Decalcified tissues of femurs were embedded in paraffin and sectioned into 5 μm slices. After the sections were deparaffinizedin xylene and rehydrated using a graded alcohol series, the sections were incubated in 3% H_2_O_2_ for 10 min and placed in 0.1% trypsin at 37 °C for 30 min for antigen retrieval. The sections were then incubated with primary antibody against Runx2 (Abcam, 1:100), HIF-1α (Abgent, 1:100), VEGF (Thermo, 1:100) or CD31 (Abcam, 1:100) overnight at 4 °C. Next, the sections were reacted with biotinylated goat anti-rabbit secondary antibody (Invitrogen) for 30 min at 37 °C. The sections were then stained with 3, 3-diaminobenzidine (Sigma) and finally counterstained with haematoxylin. The specimens were examined using a Leica image analyser at a magnification of 400×, and the results were analysed *via* computer image analysis (Leica Microsystems Ltd., UK).

### Statistical analysis

The data are expressed as the means of three experiments, each of which included triplicate or sextuplet samples for individual treatments or dosage regimens. Statistical analysis was performed using one-way ANOVA followed by Tukey’s *post hoc* test. The values are presented as the means ± SD. All statistical tests were two-sided, and the results were considered statistically significant at *P* < 0.05.

## Results

### Protective effect of SAL against the CoCl_2_-induced loss of osteoblast viability

**Figure 1:**
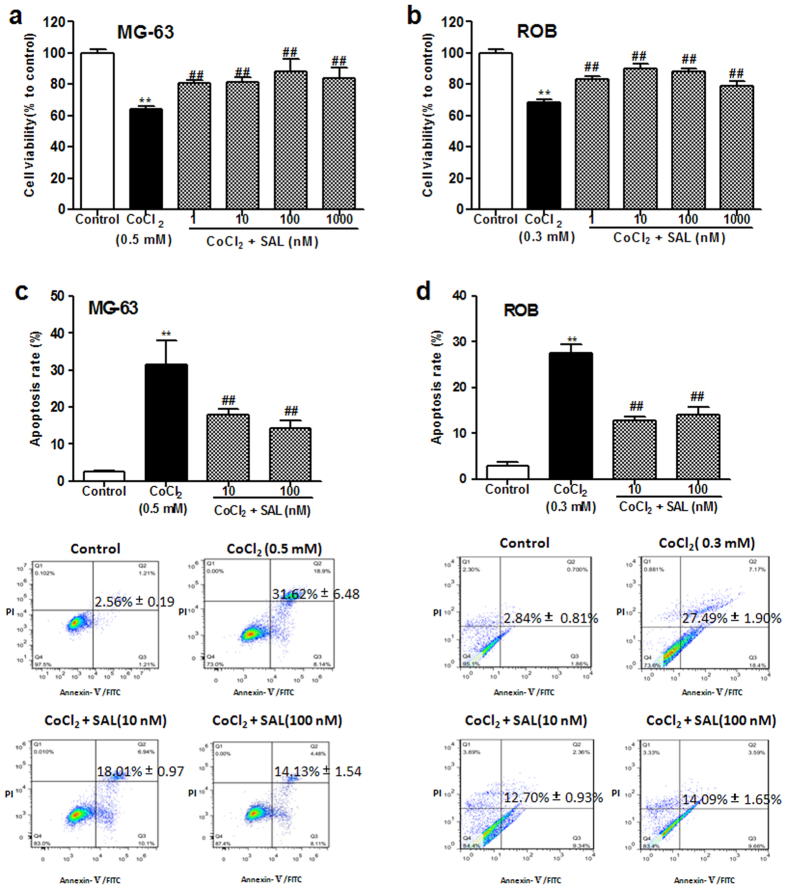
Protective effects of SAL against theCoCl_2_-induced loss of viability and increase in apoptosis ofMG-63 and ROB cells. (**a**) MG-63 cells were pre-treated with SAL (1–1000 nM) for 24 h, followed by treatment with CoCl_2_ (0.5 mM) for an additional 24 h in the presence of SAL. (**b**) ROB cells were pre-treated with SAL (1–1000 nM) for 24 h, followed by treatment with CoCl_2_ (0.3 mM) for an additional 24 h in the presence of SAL. (**c**) The mean percentage of apoptotic MG-63 cells was measured *via* flow cytometric assay. Cells were pre-treated with or without SAL for 24 h, followed by treatment with CoCl_2_ (0.5 mM) for an additional 24 h. (**d**) The mean percentage of apoptotic ROB cells was measured by flow cytometric assay. Cells were pre-treated with or without SAL for 24 h, followed by treatment with CoCl_2_ (0.3 mM) for an additional 24 h. **P < 0.01 compared with control; ^##^*P < *0.01 compared with CoCl_2_. The data are expressed as the means ± SD.

Cell viability was analysed to determine the protective effect of SAL on MG-63 and ROB cells exposed to CoCl_2_, which reduces cell viability. As shown in Fig. 1a,b, treatment with 0.5 mM CoCl_2_ for 24 h was performed to induce human osteoblastic MG-63 cell injury, and 0.3 mM CoCl_2_ was used to induce ROB cell injury in the following experiments; these treatments significantly decreased cell viability by approximately 40% compared with the control treatment. When the cells were pre-treated with SAL for 24 h prior to the addition of CoCl_2_, SAL (1–1000 nM) significantly increased the survival of the MG-63 and ROB cells compared to the corresponding cells treated with CoCl_2_ alone. These results suggested that SAL suppressed CoCl_2_-induced cytotoxicity. SAL alone was not toxic to the cells at the concentrations used in this study (data not shown).

### Protective effect of SAL against CoCl_2_-induced osteoblast apoptosis

CoCl_2_ has been shown to induce apoptosis in human alveolar macrophages, HeLa cells, and skeletal L6C5 and C2C12 cells^17^,^18^,^19^. In this study, to further confirm the ability of SAL to protect against CoCl_2_-induced cell apoptosis in osteoblast-derived cells, we stained MG-63 and ROB cells with the dyes annexin V and PI, and the stained cells were counted by flow cytometry. As shown in Fig. 1c, the percentage of apoptotic MG-63 cells increased from 2.56 to 31.62% after challenge with CoCl_2_ (0.5 mM) for 24 h. However, the percentage of apoptotic MG-63 cells was significantly reduced to 18.01% and 14.13% by pre-treatment with 10 and 100 nM SAL, respectively. Moreover, the percentage of apoptotic ROB cells increased from 2.84 to 27.49% after challenge with CoCl_2_ (0.3mM) for 24 h (Fig. 1d). However, the percentages of apoptotic ROB cells were significantly reduced to 12.70% and 14.09% by treatment with 10 and 100 nM SAL, respectively. These results demonstrated that SAL suppressed CoCl_2_-induced osteoblast apoptosis.

### Protective effect of SAL against the CoCl_2_-induced loss of osteoblast differentiation and mineralization

**Figure 2:**
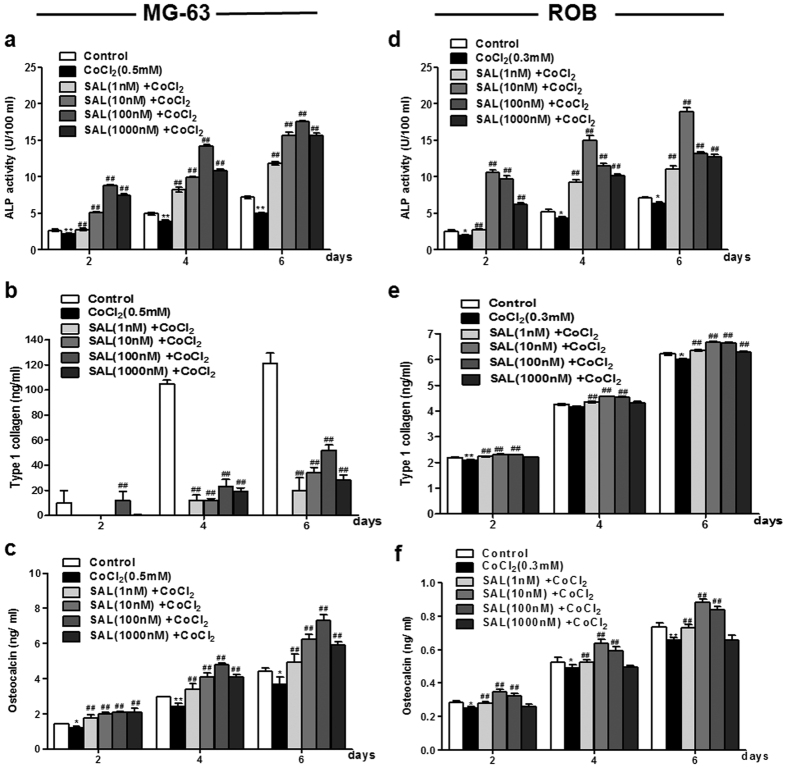
Protective effect of SAL against the CoCl_2_-induced loss of MG-63 and ROB cell differentiation. MG-63 or ROB cells were cultured in 1% FBS in the presence of either SAL (1, 10, 100 or 1000 nM) or vehicle, followed by treatment with CoCl_2_ for 2, 4, or 6 days. After SAL treatment, ALP activity was measured in MG-63 cells (**a**) or ROB cells (**d**) using an ALP activity assay kit. After SAL treatment, the level of type I collagen secretion in the cell culture supernatants of MG-63 cells (**b**) or ROB cells (**e**) was analysed by ELISA. After SAL treatment, the production of osteocalcin in the cell culture supernatants of MG-63 cells (**c**) or ROB cells (**f**) was detected by ELISA. *P < 0.05, **P < 0.01 compared with vehicle control; ^##^*P < *0.01 compared with CoCl_2_.

It is known that osteoblasts produce ALP and type I collagen, which are associated with matrix maturation and mineralization. The protective effects of SAL on the CoCl_2_-induced loss of cell differentiation were studied by determining the levels of ALP activity, collagen synthesis and osteocalcin production in MG-63 and ROB cells. ALP activity is a phenotypic marker of the early differentiation of osteoblast cells. Compared with that in the control group, ALP activity was significantly decreased in the group treated with 0.5 mM CoCl_2_. When the cells were pre-treated with SAL for 2, 4, or 6 days before the addition of CoCl_2_, ALP activity was increased for all pre-treatment periods and at all concentrations of SAL. Pre-treatment of the cells with SAL at 100 nM resulted in the most significant up-regulation of ALP activity (Fig. 2a). Furthermore, the protective effect of SAL on the terminal differentiation of osteoblasts was assessed by evaluating osteocalcin production and type I collagen synthesis. As shown in Fig. 2b, in the group treated with CoCl_2_ alone, the type I collagen level was undetectable compared with the control group. When the cells were pre-treated with SAL for 2, 4, or 6 days prior to the addition of CoCl_2_, SAL increased the protein level of type I collagen in a dose- and time-dependent manner, except for 1000 nM SAL. Similar to the level of ALP activity, the level of type I collagen was most strongly increased by SAL at a dose of 100 nM. Similar results were observed for osteocalcin secretion. SAL significantly enhanced osteocalcin secretion in a dose- and time-dependent manner compared with CoCl_2_ alone (Fig. 2c). In addition, the protective effect of SAL on the osteogenic differentiation of ROB cells was assessed. Similar trends were observed for ALP activity, although this result was most significant for the 10 nM SAL-treated group compared to the CoCl_2_-treated group (Fig. 2d). As shown in Fig. 2e, the type I collagen content was significantly increased at all concentrations of SAL. Moreover, at concentrations of 1, 10 and 100 nM, SAL significantly enhanced osteocalcin secretion (Fig. 2f).

**Figure 3:**
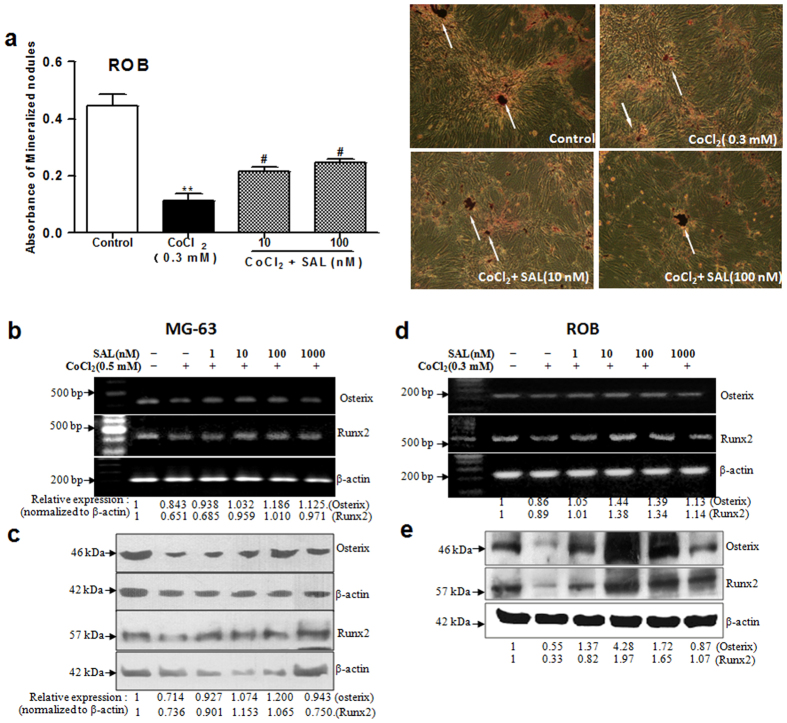
Protective effect of SAL against the CoCl_2_-induced loss of cell mineralization in ROB cells and reduction in Osterix and Runx2 expression in MG-63 and ROB cells. (**a**) SAL promoted ROB cell mineralization. After pre-treatment with either SAL (10 or 100 nM) or vehicle, followed by treatment with CoCl_2_ for 24 h, primary rat osteoblasts were induced using beta-glycerol phosphate and ascorbic acid. After the cells were cultured for 18 days, the number of mineralized bone nodules was quantified by Alizarin Red S staining (Original section, 100×).The white arrows show mineralized nodules. The absorbance of the released Alizarin Red S stain was measured at 570 nm. (**b**) SAL promoted Osterix and Runx2 mRNA expression in MG-63 cells.(**c**) SAL increased Osterix and Runx2 protein expression in MG-63 cells. (**d**) SAL promoted Osterix and Runx2 mRNA expression in ROB cells. (**e**) SAL increased Osterix and Runx2 protein expression in ROB cells. MG-63 or ROB cells were cultured in 1% FBS in the presence of either SAL (1, 10, 100 or 1000 nM) or vehicle, followed by treatment with CoCl_2_ for 48 h. The mRNA and protein levels of Osterix and Runx2 were examined by semi-quantitative RT-PCR and Western blot. **P < 0.01 compared with vehicle control; ^#^*P < *0.05 compared with CoCl_2_.

We further investigated the effect of SAL on the mineralization of ROB cells. For matrix calcification, the absorbance of the released Alizarin Red S stain was measured at a wavelength of 570 nm. After 18 days in culture, CoCl_2_ (0.3 mM) treatment significantly decreased the number of mineralized nodules stained by Alizarin Red S compared with the control treatment. When the cells were pre-treated with SAL for 24 h before the addition of CoCl_2_, SAL (10 or 100 nM) significantly increased the number of mineralized nodules of ROB cells compared to CoCl_2_ alone (Fig. 3a). Our results demonstrated that SAL attenuated the loss of osteoblast differentiation and mineralization induced by CoCl_2_.

### SAL protects against the CoCl_2_-induced loss of cell differentiation by increasing the expression of Osterix and Runx2

The transcription factors Runx2 and Osterix play a critical role in osteoblastic differentiation and osteogenesis. As mentioned above, SAL significantly ameliorated the CoCl_2_-induced loss of osteoblastic differentiation by increasing ALP activity, osteocalcin production and type I collagen synthesis. Thus, we further analysed the effect of SAL on the expression of Runx2 and Osterix in a hypoxic environment. Compared with the control treatment, CoCl_2_ treatment down-regulated Runx2 and Osterix expression at the mRNA and protein levels in MG-63 and ROB cells. However, following pre-treatment with SAL, Runx2 and Osterix expression was up-regulated at the mRNA and protein levels in MG-63 cells (Fig. 3b,c) and ROB cells (Fig. 3d,e). Runx2 is expressed during the early stages of osteochondroprogenitorfate determination; Osterix induction then follows during osteoblast maturation. Our data showed that SAL promotes osteoblastic differentiation and maturation by increasing the expression of Osterix and Runx2 during osteogenesis in a hypoxic environment.

### SAL promotes VEGF production by enhancing the transactivation of HIF-1α in a hypoxic environment

**Figure 4:**
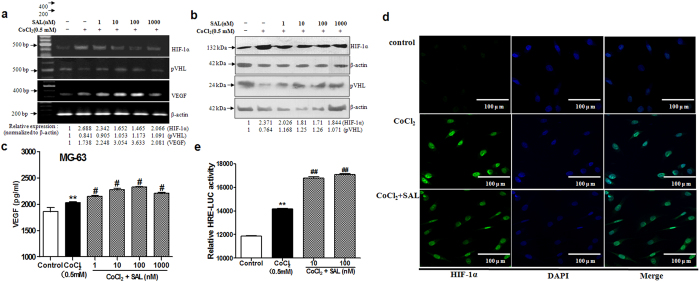
Effects of SAL on components of the HIF-1α pathway in MG-63 cells in a hypoxic environment. (**a**) Detection of the mRNA levels of HIF-1α, pVHL and VEGF by semi-quantitative RT-PCR. (**b**) Western blot analysis of HIF-1α and pVHL protein expression. The numbers below the lanes indicate the levels of mRNA or protein expression compared with the control levels. (**c**) The level of VEGF secretion into the cell culture supernatants was analysed by ELISA. (**d**) Effect of SAL on the transcriptional activity of HIF-1α. The relative luciferase activity level was measured and normalized to the enzymatic activity level of β-galactosidase. (**e**) Effect of SAL on the translocation of HIF-1α. After culturing in the presence of 100 nM SAL or vehicle followed by treatment with CoCl_2_ for 24 h, MG-63 cells were treated as described in the Methods section. Then, the nuclear localization of HIF-1α was observed *via* confocal microscopy. The position of the cell nucleus was confirmed by staining with DAPI (blue), and HIF-1α expression was detected as FITC staining (green) (Original section, 400×). **P < 0.01 compared with vehicle control; ^#^*P < *0.05, ^##^*P < *0.01 compared with CoCl_2_.

The HIF-1α pathway is the central regulator of adaptive responses to low oxygen availability and is required for normal skeletal development. We next examined whether SAL regulates the HIF-1α pathway. The levels of intracellular pVHL, HIF-1α and VEGF were measured by Western blot analysis and RT-PCR analysis as described in the Methods section. The level of HIF-1α, a key hypoxia-related effector that participates in the regulation of the expression of many genes and proteins after hypoxic stimulation, was markedly increased after CoCl_2_ challenge for 24 h (Fig. 4a,b). Moreover, the mRNA and protein expression levels of the HIF-1α target gene VEGF were up-regulated by treatment with CoCl_2_. In contrast to HIF-1α and VEGF, pVHL, the HIF-1α-recognizing subunit of a ubiquitin ligase that promotes HIF-1α ubiquitination and proteasomal degradation, was down-regulated at the mRNA and protein levels in CoCl_2_-treated cells (Fig. 4a,b). However, the excessive accumulation of HIF-1α was attenuated by pre-treatment with SAL in the concentration range from 1 to 1000 nM. Furthermore, the HIF-1α target gene VEGF was up-regulated by pre-treatment with SAL (1–1000 nM) (Fig. 4c).

Nuclear translocation of HIF-1α is necessary for the transcriptional activation of a variety of HIF-1α–regulated genes, including VEGF. We next analysed whether SAL influenced the nuclear translocation of HIF-1α in MG-63 cells in a hypoxic environment. Confocal microscopic analysis showed that exposure of MG-63 cells to CoCl_2_ resulted in the translocation of HIF-1α from the cytoplasm to the nucleus; this nuclear translocation was more complete in MG-63 cells treated with CoCl_2_ alone than in SAL-pre-treated MG-63 cells (Fig. 4d).

The classical genomic functions of HIF-1α are initiated by its binding to the nuclear HIF-1β, followed by dimerization and subsequent binding to HREs. Therefore, we next evaluated the effect of SAL on a classic HRE in MG-63 cells. MG-63 cells containing endogenous HIF-1α were transiently transfected with an HRE-luciferase reporter construct and then treated with CoCl_2_ (0.5 mM), with or without SAL (10 or 100 nM). As expected, both CoCl_2_ and SAL strongly enhanced transcription derived from the HRE reporter (Fig. 4e).

Collectively, these results indicated that in a hypoxic environment, SAL dramatically up-regulated the expression of VEGF, a well-known downstream target of HIF-1α. Despite its functions in down-regulating HIF-1α protein expression and nuclear translocation, SAL promotes the transcriptional activation of HIF-1α and affects VEGF expression.

### Effects of SAL on the serum levels of minerals, ALP, BGP and E_2_ in OVX rats

**Figure 5:**
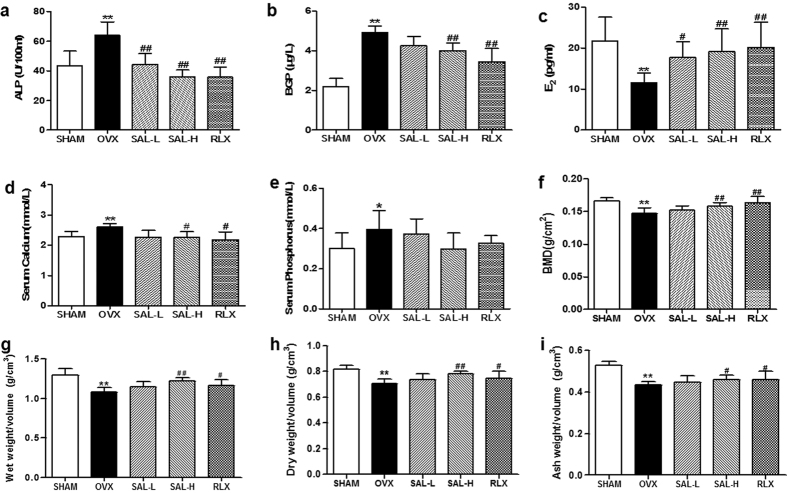
Effects of SAL on the serum levels of minerals and bone biochemical markers, bone mineral density (BMD) and bone weight in OVX rats. (**a**) ALP. (**b**) BGP. (**c**) E_2_. (**d**) Serum Ca. (**e**) Serum P. (**f**) BMD. (**g**) Wet weight/volume. (**h**) Dry weight/volume. (**i**) Ash weight/volume. The data are presented as themeans ± SD (n = 9 per group). **P < 0.01 compared with the sham control group; ^#^*P < *0.05, ^##^*P < *0.01 compared with the OVX group. The OVX model was established in female Wistar rats as described in the Methods section. After treatment with SAL or RLX, the rats were sacrificed at 12 weeks after surgery.

After showing that SAL plays critical roles in osteoblast-mediated bone formation and HIF-1α-dependent VEGF expression, we were interested in assessing its effects *in vivo*. The validity of our osteoporosis model was confirmed by examining the serum levels of ALP, BGP and E_2_ at twelve weeks after ovariectomy. Compared with the control group, the OVX group displayed significantly increased serum ALP and BGP levels. As shown in Fig. 5a,b, the serum ALP and BGP levels in the rats in the SAL-H and RLX groups were significantly decreased after twelve weeks of treatment compared with those in the model group. In contrast, SAL increased serum E_2_ levels in the OVX rats, with a maximum effect that approximated the effect of RLX (Fig. 5c). Moreover, serum mineral levels were monitored for changes that may affect bone mineralization. The serum levels of minerals (Ca and P) in each group are shown as percentages. The results indicated that the serum Ca levels in the SAL-H and RLX groups were significantly lower than were those in the OVX group (Fig. 5d). However, the serum P levels did not differ between the model group and the SAL groups (Fig. 5e).

### Effects of SAL on BMD and bone weight in OVX rats

The validity of the osteoporosis model was also confirmed by examining BMD at twelve weeks after ovariectomy. Compared with the BMD of the rats in the control group, significantly decreased BMD was found in the right femurs of the rats in the OVX group. However, compared with the model group, the SAL-H and RLX groups displayed significantly increased BMD (Fig. 5f). Similar effects of SAL were also observed for the wet weight, dry weight and ash weight of femurs, as these parameters were significantly higher in the SAL-H and RLX groups than in the model group (Fig. 5g–i). Taken together, these data indicated that SAL might contribute to the alleviation of osteoporosis.

### Protective effect of SAL against bone loss in OVX rats

**Figure 6:**
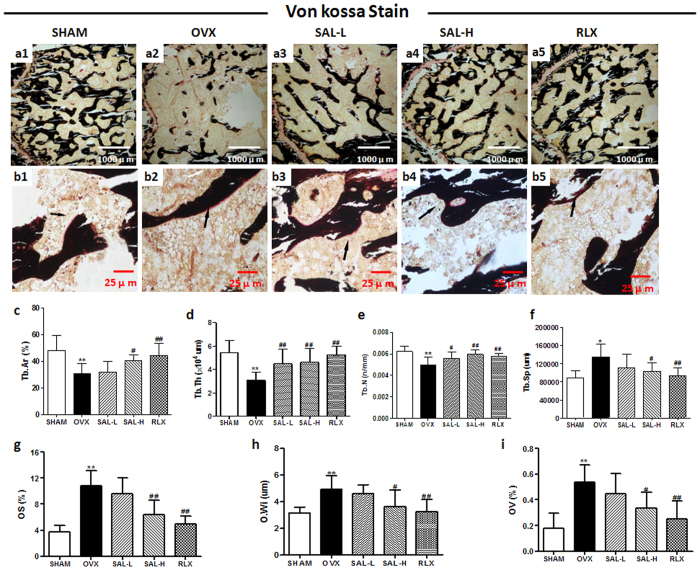
Effects of SAL on the histomorphology of the proximal femur in OVX rats. Von Kossa staining of femurs (a1–a5:40×; b1–b5:400×). The black arrows show osteoids of the proximal femur. (**c**) Percent trabecular area (Tb.Ar). (**d**) Trabecular thickness (Tb.Th). (**e**) Trabecular separation (Tb.Sp). (**f**) Trabecular number (Tb.N). (**g**) Osteoid surfaces (OS). (**h**) Osteoid width (O.Wi). (**i**) Osteoid volume (OV).The data are expressed as the means ± SD (n = 9 per group). **P < 0.01 compared with the sham control group; ^#^*P* < 0.05, ^##^*P* < 0.01 compared with the OVX group.

We administered SAL as early as 7 days after ovariectomy to evaluate its effects on trabecular bone microarchitecture. We sacrificed all animals 12 weeks after the operation. No significant difference in body weight between the five groups of rats was observed. Analysis of the properties of trabecular bone in the distal femoral metaphyses indicated that ovariectomy induced the deterioration of trabecular bone microarchitecture in the rats, as demonstrated by the reduction in Tb.Ar, Tb.N and Tb.Th compared with the sham operation (Fig. 6a1–a5,c–e). In contrast, as shown in Fig. 6f, Tb.Sp was significantly increased following ovariectomy. However, treating OVX rats with a high dose of SAL at 20 mg/kg significantly reversed the ovariectomy-induced changes in these parameters and maintained the microarchitecture of trabecular bone in the distal femoral metaphyses. SAL at 5 mg/kg alleviated the trabecular bone mass loss and microarchitecture deterioration but did not significantly alter Tb.Ar or Tb.Sp. These increments in trabecular bone parameters were readily observable *via* von Kossa staining. Osteoid formation in bone is prevalent during the treatment of osteoporosis. We quantified micromorphological parameters of osteoid, including OS, O.Wi and OV. Compared with the sham group, the OVX group displayed increases in these three parameters. However, SAL inhibited these deleterious effects, as demonstrated by a decrease in these parameters in the SAL-H and RLX groups (Fig. 6b1–b5,g–i).

**Figure 7:**
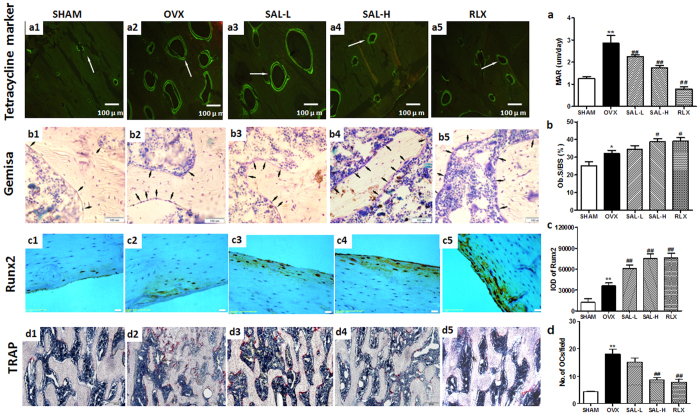
Effects of SAL on the histomorphology of the proximal femur in OVX rats. (a1–a5, **a**) Double labelling in the endplate region was used to calculate the mineral apposition rate (MAR) in each femur region (100×). The white arrows show tetracycline double labelling of the femur region. (b1–b5, **b**) Giemsa staining of the femur was used to calculate the osteoblast absorption surfaces (Ob.S). The black arrows show osteoblasts. (c1–c5, **c**) Microscopic images of sections stained with immunohistochemisty of Runx2 antibody (200×) and effects of SAL on Runx2 expression in OVX rats. (d1–d5, **d**) Microscopic images of tartrate-resistant acid phosphatase (TRAP) staining femur section (100×) and the statistical result of number of osteoclasts. The data are expressed as the means ± SD (n = 9 per group). **P < 0.01 compared with the sham control group; ^#^*P < *0.05, ^##^*P < *0.01 compared with the OVX group.

In addition, the MAR was calculated for each femur region *via* double labelling. A significant increase in the MAR was observed in the OVX group compared with the sham group. However, compared with model group, the SAL and RLX groups displayed significant decreases in the MAR (Fig. a1–a5,a). Furthermore, based on Giemsa staining, Ob.S was observable; significant increases in Ob.S were observed in the OVX group compared with the sham group. Alternatively, compared with model group, the SAL-H and RLX groups exhibited significantly increased Ob.S (Fig. 7b1–b5, b). As a master osteoblast transcription factor, Runx2 was examined with immunohistochemistry. The expression of Runx2 was increased in the OVX group compared with the sham group, which were further enhanced by SAL and RLX (Fig. 7c1–c5, c). and Tartrate-resistant acid phosphatase (TRAP) staining revealed that the number of multinucleated osteoclasts was increased in the OVX group and obviously decreased by high-dose SAL and RLX (Fig. 7d1–d5, d).

**Figure 8:**
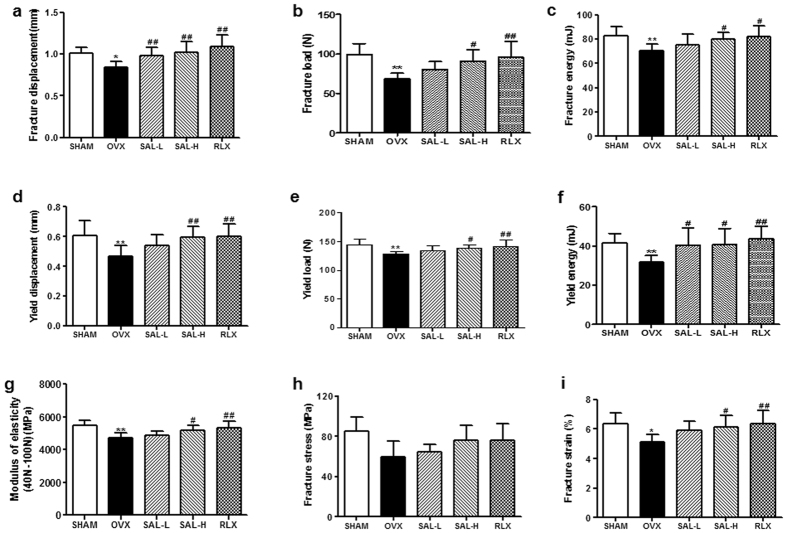
Effects of SAL on the biomechanical parameters of femurs in OVX rats. (**a–f**) Structural mechanics (fracture displacement, fracture load, fracture energy, yield displacement, yield load, and yield energy). (**g–i**) Material mechanics (modulus of elasticity, fracture stress, and fracture strain). The data are expressed as the means ± SD (n = 9 per group). **P < 0.01 compared with the sham control group; ^#^*P < *0.05, ^##^*P < *0.01 compared with the OVX group.

Next, we evaluated the mechanical properties of the femur *via* a three-point bending test using an INSTRON 5865; the results for the mechanical parameters are shown in Fig. 8. A significant reduction in femur mechanical function, as demonstrated by decreases in parameters related to structural mechanics (fracture displacement, fracture load, fracture energy, yield displacement, yield load, and yield energy) (Fig. 8a–f) and parameters related to material mechanics (modulus of elasticity, fracture stress and fracture strain)(Fig. 8g–i), was detected in the OVX group compared with the sham group. In contrast, improved mechanical function was observed in the SAL and RLX groups compared with the OVX group. RLX, used as a positive control, has been shown to enhance bone regeneration *in vivo*. Therefore, the data described above revealed that SAL possesses similar activities to those of RLX and exerts a dramatically osteoprotective effect in OVX rats.

**Figure 9:**
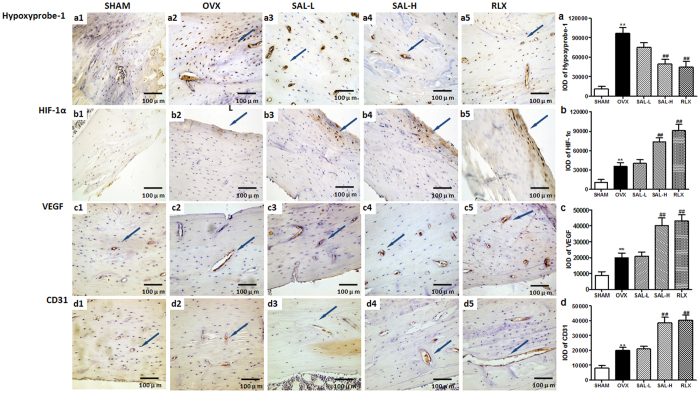
Effects of SAL on Hypoxyprobe-1 staining intensity and HIF-1α, VEGF and CD31 expression in OVX rats. Representative sections stained with Hypoxyprobe-1 or antibodies against HIF-1α, VEGF and CD31 are shown (the blue arrows, 400×). Quantitative measurements of the staining intensity of Hypoxyprobe-1 (a1–a5, **a**) and the expression levels of HIF-1α (b1–b5, **b**), VEGF (c1–c5, **c**) and CD31 (d1–d5, **d**) are shown. The data are presented as the means ± SD. **P < 0.01 compared with the sham group; ^##^*P < *0.01 compared with the OVX group.

Immunohistochemistry was used to further assess the influence of SAL on the HIF-1α pathway. At 12 weeks after ovariectomy surgery, positive Hypoxyprobe-1staining was detected within the bone tissue of the OVX group. These data illustrated that hypoxia was present within the bone tissue of the OVX rats (Fig. 9a1–a5,a). Moreover, positive HIF-1α staining was evident within the bone tissue of the OVX group. Compared with the OVX group, the SAL-H and RLX groups displayed significantly increased HIF-1α staining. However, no difference in HIF-1α staining was detected between the OVX group and the SAL-L group (Fig. 9b1–b5,b). Consistent with the HIF-1α staining results, increased expression of VEGF was observed in the SAL-H and RLX groups compared to that in the OVX group (Fig. 9c1–c5,c). VEGF is known to act as a potent activator of angiogenesis. Thus, we next examined the expression level of the endothelial cell-specific marker CD31. As shown in Fig. 9d1–d, bone tissue stained with anti-CD31 antibody revealed that SAL significantly increased the formation of new blood vessels. Collectively, our *in vivo* data indicated that SAL, especially high-dose SAL, coupled bone formation with HIF-1α-VEGF-induced angiogenesis, thereby preventing bone loss in OVX rats.

## Discussion

In the present study, we have demonstrated that in a hypoxic environment, SAL enhanced osteogenesis by stimulating cell viability, differentiation, and mineralization and by up-regulating Osterix and Runx2 expression. These effects were attributed to the activation of HIF-1α signalling pathways. Moreover, our *in vivo* results showed that SAL ameliorated OVX-induced osteoporosis. In addition, significant remediation of angiogenesis, resulting from increases in VEGF expression, also led to improved bone health in OVX rats. To our knowledge, this report is the first to demonstrate the unambiguous osteoporosis-preventive effect of SAL against CoCl_2_-induced injury.

The process of bone formation is reported to include initial osteoblast viability followed by increased ALP activity, the development and maturation of the extracellular matrix and, ultimately, mineralization^20^. Moreover, the activities of ALP (a marker of cell maturation and mineralization) and osteocalcin (a marker of terminal differentiation) are important for the regulation of bone matrix mineralization in osteoblasts^21^,^22^. SAL-induced increases in ALP activity and mineralization in MC3T3-E1 cells were also observed by Zhang *et al*. As they did not find any evidence of a direct effect of SAL on osteoblastic cells, the authors suggested that the prevention of bone loss by SAL is largely mediated by the inhibition of oxidative damage to bone-forming cells and the release of bone-resorbing mediators^23^. Our *in vitro* data showed that SAL promoted the viability and differentiation of both MG63 and ROB cells in an *in vitro* CoCl_2_-induced hypoxia model, as demonstrated by the increases in ALP activity, collagen synthesis and osteocalcin production. Consistently, our data demonstrated that following SAL treatment, the expression of the transcription factors Runx2 and Osterix, which are key regulators in osteoblast differentiation, was significantly up-regulated in both osteoblastic cell types. Runx2 and Osterix are considered master osteogenic factors because their respective null mice do not form mature osteoblasts^24^,^25^. Recent results have indicated that Runx2 and Osterix act in a collaborative manner to induce osteogenic genes involved in bone matrix formation^26^,^27^. In addition, previous studies revealed that hypoxia inhibits the expression of Runx2, down-regulating Runx2 through the HIF-1α-TWIST pathway^28^. Runx2 has been previously reported to enhance the stability and transcriptional activity of HIF-1α by interacting with the oxygen-dependent degradation domain and competing with PVHL to inhibit ubiquitination^29^. Such results, in concert with other studies and ours, suggest a relationship between Runx2 and HIF-1α. SAL may affect the maturation and differentiation of osteoblasts at various levels while regulating the complex process of HIF-1α activation in an *in vitro* hypoxia model.

Hypoxia is a strong stimulus that stabilizes HIF-1α expression by inhibiting its ubiquitination and degradation^30^. HIF-1α accumulates under hypoxic conditions and acts as a cellular oxygen sensor to mediate diverse subsequent molecular and cellular events^31^. As an extensively used hypoxia mimic, CoCl_2_ can induce the accumulation of HIF-1α^32^,^33^,^34^. Therefore, we used a CoCl_2_-induced hypoxia model in the present study. Some reports have shown that the HIF-1α pathway is critical for coupling angiogenesis to osteogenesis during long bone formation. The activation of HIF-1α in osteoblasts *via* the disruption of its degradation pathway produced robust bone modelling early in development^7^. Consistent with the “promoting osteogenesis by enhancing angiogenesis” theory, HIF-1α pathway activators (DMOG or DFO) or mesenchymal stem cells overexpressing HIF-1α have been shown to improve fracture and bone defect healing^35^,^36^,^37^,^38^,^39^,^40^,^41^. HIF-1α that binds to the aryl hydrocarbon receptor nuclear translocator (ARNT) is known as HIF-1β. During hypoxia, prolyl hydroxylation is blocked, leading to HIF-1α stabilization, subsequent nuclear import, and dimerization with HIF-1β, which initiates the transcription of HIF-responsive genes^42^. In this report, we have shown that in spite of the function of SAL in down-regulating HIF-1α protein expression and nuclear translocation, SAL promotes the transcriptional activation of HIF-1α. We speculated the possibility that SAL accelerates the binding of HIF-1α and HIF-1β, resulting in enhancement of transcriptional activity and dramatic up-regulation of VEGF expression in a hypoxic environment.

Impaired regulation of HIF-1α is also observed in other pathological processes, such as the development of diabetic wounds, and the stabilization of HIF-1α is pivotal for reversing these pathological processes^43^. Mice in which HIF-1α signalling was activated developed extremely dense, heavily vascularized bones, and these mice were protected from ovariectomy-or age-induced bone loss^10^,^44^. More importantly, age-related bone loss has been prevented by the administration of DFO, ahypoxiamimic^45^. Therefore, the stabilization of HIF-1α might also help prevent oestrogen deficiency-induced bone loss.

Recently, Zhang JK reported that SAL prevented bone loss and trabecular microarchitecture deterioration in an ovariectomy-induced osteoporosis mouse model^23^. Bone quality is determined by the microarchitectural, geometric and material properties of the bone. Measuring microarchitectural parameters such as BV/TV, Tb.N, Tb.Sp, Tb.Th, Conn.D, SMI and BMD may improve our ability to estimate bone strength^46^,^47^. The effects of ovariectomy on bone are smaller in cortical compartments than in trabecular compartments^48^. Therefore, we observed the effect of SAL on the trabecular microarchitecture. All parameters examined, including BMD, Tb.Ar, Tb.Th, Tb.N, and Tb.Sp, showed that treatment with 20 mg/kg SAL for 3 months ameliorated the ovariectomy-induced reductions in bone structural indices, BMD and trabecular thickness. Furthermore, histological examination showed that ovariectomy exerted negative effects on the trabecular bone microarchitecture. However, this effect was partially reversed by supplementation with SAL. Some reports have shown that RLX protected against osteoporosisin OVX mice and in postmenopausal arthritis^49^,^50^,^51^,^52^,^53^. In this study, we observed that SAL possessed *in vivo* therapeutic effects similar to those of RLX. Many factors contribute to osteoporosis, such as oestrogen deficiency, hereditary, nutritional deficiencies, chronic diseases and ageing. Oestrogen deficiency caused by menopause contributes to increased bone turnover and osteoclastic resorption, exceeding the rate of osteoblastic formation and resulting in a loss of bone mass and decreased bone strength in women^54^. E_2_ levels in the model group were significantly decreased, but SAL reversed serum E_2_ levels in the OVX rats. These data suggested that SAL might significantly increase trabecular bone mass in the OVX rat model and might exert a preventive effect against oestrogen deficiency-induced osteoporosis. In addition, SAL has been previously reported to stimulate insulin secretion and improve insulin sensitivity, which can enhance the leptin on bone metabolism in the diabetic rats with osteoporosis^55^. Furthermore, SAL has been reported to increase serum testosterone level in tail stress mice^56^. **T**hese results suggested that SAL may be effect the endocrine system in stress. Therefore, we speculated the possibility that the up-regulation of serum E_2_ levels by SAL may be related to affect the other endocrine tissues, such as the adrenal cortex. Further study is needed to clarify the role of SAL on E_2_ levels in OVX rats.

Osteoclasts play a key role in the femur resorption associated with osteoporosis. The present study identified obvious bone resorption in the OVX group, whereas the SAL-treated groups showed a significant and dose-dependent suppression of bone loss. The histological analysis of tissues stained with TRAP further supported these results because they revealed that SAL reduced the number of TRAP-positive multinucleated osteoclasts. Therefore, the inhibition of osteoclast formation and function may be the key targets for therapeutic agents in the treatment of OVX-induced osteoporosis.

Given that angiogenesis and osteogenesis are closely coupled, multiple studies have investigated the role of angiogenesis in the pathogenesis of osteoporosis. Accumulating evidence from studies in cells, animals, and patients indicates that low oestrogen levels contribute to fewer vessels or decreases in the levels of angiogenic factors, resulting in the progression of osteoporosis^3^,^57^,^58^,^59^,^60^,^61^,^62^,^63^. The findings of the current study support this concept. Ovariectomy increased the expression of HIF-1α, VEGF and CD31 in bone samples. VEGF, the best known and most critical angiogenic factor, induces vessel formation and regulates the balance between osteogenic and adipogenic differentiation in MSCs. Mice harbouring VEGF deficiency in osteoblastic precursor cells exhibit an osteoporosis-like phenotype characterized by reduced bone mass and increased bone marrow fat content^64^. Thus, normalization of low VEGF levels in postmenopausal women might be of critical significance. As expected, SAL administration increased the expression of HIF-1α, VEGF and CD31 in trabecular bone. These findings strongly suggested that SAL prevents bone loss and improves bone quality in OVX rats *via* angiogenesis-osteogenesis coupling.

From the results of our current study, we observed that SAL has opposite effects on bone mineralization and HIF-1α expression *in vitro* and *in vivo*. The formation of a mineralized nodule is a definitive hallmark of osteoblastic differentiation. Our results suggested that SAL treatment accelerated ROB cell differentiation by increasing mineralization *in vitro* (Fig. 3a). However, the MAR (Fig. 7a) is commonly used to calculate the mean rate of bone formation *in vivo*. The MAR is obtained by measuring the average of the vertical distance between two tetracycline fluorescence labels and the time of the interval mark in animals. MAR is one of bone turnover parameters. Some reports have reported that, at three months after OVX surgery, osteoporotic state with increased bone turnover was confirmed by bone histomorphometry. Specifically, OVX induced significantly increased MAR which caused further deterioration. However, minodronic acid and dried plum suppressed OVX-induced increases in bone turnover at the tissue level, thereby improving the deterioration of bone quality under osteoporotic disease conditions^65^,^66^. In our study, we observed that SAL possessed the similar results *in vivo*. In addition, our *in vivo* results showed that SAL significantly increased the expression levels of HIF-1α and VEGF within the femur. Previous *in vitro* studies have shown that SAL only promoted the transcriptional activation of HIF-1α and dramatically up-regulated VEGF expression in a hypoxic environment. Based on the above results, we are not able to exclude the possibility of other correlative elements. Hypoxia is prominent in the microenvironment in both bony and soft tissue injury. The bone lining cells, which include osteoblasts, osteoclasts, and endosteal and periosteal lining cells, are all involved in the repair process *in vivo*. Thus, the anti-anoxic functions of SAL in this model may differ from the effects of SAL on cells.

In conclusion, our studies demonstrated that SAL protected MG63 and ROB cells from CoCl_2_-induced osteoblast damage and dysfunction by suppressing the HIF-1α signalling pathway. Moreover, SAL prevented bone loss and trabecular microarchitecture deterioration in an ovariectomy-induced osteoporosis rat model. Our results suggested that SAL may serve as a good candidate for preventing and treating osteoporosis associated with the activation of HIF-1α-VEGF signalling pathways.

## Additional Information

**How to cite this article**: Li, L. *et al*. Protective effect of Salidroside against bone loss *via* hypoxia-inducible factor-1α pathway-induced angiogenesis. *Sci. Rep.* **6**, 32131; doi: 10.1038/srep32131 (2016).
